# Performance of Node-RADS Scoring System for a Standardized Assessment of Regional Lymph Nodes in Bladder Cancer Patients

**DOI:** 10.3390/cancers15030580

**Published:** 2023-01-18

**Authors:** Costantino Leonardo, Rocco Simone Flammia, Sara Lucciola, Flavia Proietti, Martina Pecoraro, Bruno Bucca, Leslie Claire Licari, Antonella Borrelli, Eugenio Bologna, Nicholas Landini, Maurizio Del Monte, Benjamin I. Chung, Carlo Catalano, Fabio Massimo Magliocca, Ettore De Berardinis, Francesco Del Giudice, Valeria Panebianco

**Affiliations:** 1 Department of Maternal Infant and Urological Sciences, Sapienza University Rome, Policlinico Umberto I Hospital, 00161 Rome, Italy; 2 Department of Radiological Sciences, Oncology and Pathology, Sapienza University, 00161 Rome, Italy; 3 Department of Urology, Standford University School of Medicine, Standford, CA 94305, USA; 4 Department of Anatomopathological, Oncology and Pathology, Sapienza University, 00161 Rome, Italy

**Keywords:** Node-RADS, bladder cancer, radical cystectomy, lymph-node invasion, bladder cancer staging

## Abstract

**Simple Summary:**

Node-RADS represents a five-item comprehensive score, based on both size and configuration criteria, to standardize the radiologic assessment of lymph node invasion. In bladder cancer patients, pelvic lymph node staging is of utmost importance in clinical decision-making. In the current study, we applied Node-RADS scoring for a pelvic lymph-node evaluation of bladder cancer patients. We concluded that the Node-RADS score exhibits a moderate-to-high overall accuracy for identification of lymph node invasion, with the possibility of setting different cut-off values according to specific clinical scenarios. However, these results need to be validated on larger cohorts before drawing definitive conclusions.

**Abstract:**

Background: Current cross-sectional imaging modalities exhibit heterogenous diagnostic performances for the detection of a lymph node invasion (LNI) in bladder cancer (BCa) patients. Recently, the Node-RADS score was introduced to provide a standardized comprehensive evaluation of LNI, based on a five-item Likert scale accounting for both size and configuration criteria. In the current study, we hypothesized that the Node-RADS score accurately predicts the LNI and tested its diagnostic performance. Methods: We retrospectively reviewed BCa patients treated with radical cystectomy (RC) and bilateral extended pelvic lymph node dissection, from January 2019 to June 2022. Patients receiving preoperative systemic chemotherapy were excluded. A logistic regression analysis tested the correlation between the Node-RADS score and LNI both at patient and lymph-node level. The ROC curves and the AUC depicted the overall diagnostic performance. In addition, the sensitivity, specificity, positive predictive value (PPV), and negative predictive value (NPV) were calculated for different cut-off values (>1, >2, >3, >4). Results: Overall, data from 49 patients were collected. Node-RADS assigned on CT scans images, was found to independently predict the LNI after an adjusted multivariable regression analysis, both at the patient (OR 3.36, 95%CI 1.68–9.40, *p* = 0.004) and lymph node (OR 5.18, 95%CI 3.39–8.64, *p* < 0.001) levels. Node-RADS exhibited an AUC of 0.87 and 0.91 at the patient and lymph node levels, respectively. With increasing Node-RADS cut-off values, the specificity and PPV increased from 57.1 to 97.1% and from 48.3 to 83.3%, respectively. Conversely, the sensitivity and NPV decreased from 100 to 35.7% and from 100 to 79.1%, respectively. Similar trends were recorded at the lymph node level. Potentially, Node-RADS > 2 could be considered as the best cut-off value due to balanced values at both the patient (77.1 and 78.6%, respectively) and lymph node levels (82.4 and 93.4%, respectively). Conclusions: The current study lays the foundation for the introduction of Node-RADS for the regional lymph-node evaluation in BCa patients. Interestingly, the Node-RADS score exhibited a moderate-to-high overall accuracy for the identification of LNI, with the possibility of setting different cut-off values according to specific clinical scenarios. However, these results need to be validated on larger cohorts before drawing definitive conclusions.

## 1. Introduction

In patients with bladder cancer (BCa), utilizing the tumor, node, metastasis (TNM) classification is strongly recommended to guide treatment and determine prognosis [1,2]. Specifically, the local tumor stage (T stage) can be accurately assessed, based on a transurethral resection of bladder tumors (TURBTs) and magnetic resonance imaging (MRI) for the definition of muscle-invasive bladder cancer and extravesical extension [3,4]. Conversely, the preoperative assessment of the regional node stage (clinical N stage) relies exclusively on traditional cross-sectional imaging modalities, such as computed tomography (CT) or MRI, which adopts non-standardized criteria to define nodal involvement [3]. This lack of standardization has shown a poor predictive performance of imaging, compared to pathology reports in BCa, as well as in other oncologic pathologies [5]. Nonetheless, the clinical N stage (cN) is of utmost importance for the management decision making. Indeed, the 8th edition TNM claimed that cN positive BCa patients would be no longer considered as stage IV disease, based on a better prognosis, in comparison to patients with distant metastasis [6]. Moreover, European and North American guidelines recommend neoadjuvant chemotherapy followed by radical cystectomy (RC) for cN negative muscle-invasive BCa patients, based on results from randomized clinical trials [7,8,9]. Conversely, management of cN positive BCa patients mainly relied on retrospective studies [10,11,12,13,14,15,16,17,18], suggesting systemic chemotherapy with the addition of consolidative local therapy, in case of a favorable response [1,2]. Under such premises, an accurate and standardized assessment of the cN stage is strongly required to deliver optimal treatment and adequate counseling. Interestingly, Elsholtz et al. proposed a comprehensive score, based on both size and configuration criteria, namely Node-RADS, to standardize the radiologic assessment of the lymph node invasion (LNI) on CT and MRI scans, which could be applied for any type of tumors, at any anatomical site, as well as to regional and non-regional lymph nodes [19]. Since its introduction in 2021, Node-RADS has been validated only in prostate cancer, showing promising results [20]. However, no previous reports have investigated its role in BCa. To address this void, we reviewed preoperative CT scans of BCa patients treated with RC at our institution. We hypothesized that a higher Node-RADS score is associated with increased risk of LNI and we tested the overall diagnostic performance of Node-RADS scoring.

## 2. Materials and Methods

### 2.1. Study Design and Patient Population

For the current pilot study, we relied on our prospectively maintained database of BCa patients treated with radical cystectomy (RC) at our institution (Sapienza University of Rome, Policlinico Umberto I), from which the data were retrospectively collected. We included all patients with a diagnosis of either muscle-invasive BCa or high-risk non-muscle-invasive BCa, unresponsive to intravesical therapies, treated with RC plus bilateral pelvic lymph node dissection (PLND), from January 2019 to June 2022. Patients receiving preoperative systemic chemotherapy, patients for whom a preoperative CT scan was not available, patients who did not receive an extended PLND, patients who did not have a packeted lymph node submission, and patients with missing data were excluded.

### 2.2. Baseline Variables

For each patient the following data were retrieved from our prospectively maintained database: age at surgery, clinical T staging (cT), tumor grade (WHO 2004), presence of lymphovascular invasion (LVI), concomitant carcinoma in situ (CIS) at last TURBT, type of surgery (open vs. minimally invasive), number of lymph-nodes removed, pathologic T (pT), N stage (pN), and surgical margin status (SRM) at RC. Clinical T stage was divided between organ-confined (OC, cT ≤ 2) vs. non-organ confined (NOC, cT > 2).

### 2.3. CT Scan Examination and Node-RADS Assessment

**Figure 1:**
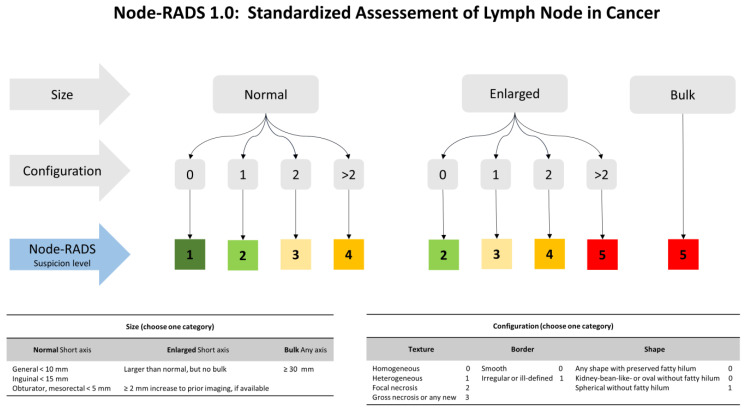
Explanation of the Node-RADS scoring system, adapted from the original publication [19].

CT scan images were acquired using two multidetector CT scanners (Somatom Sensation 128, Somatom Sensation 64; Siemens Healthcare (Victoria, Australia)). The acquisition parameters used were those recommended by the manufacturer. Specifically, the baseline, arterial, nephrographic, and excretory phases of the abdomen and pelvis were acquired for the evaluation of the entire excretory tract. Multiplanar (axial, coronal, and sagittal) images were reconstructed with a slice thickness of 1 mm, using the classical filtered back-projection method with a soft tissue kernel of B20 and a bone kernel of B60. CT images were retrospectively evaluated by a fellow radiologist (VP) with 15-years’ experience in the field of CT imaging, as well as in the genito-urinary field. The reader was blinded to the post-operative pathological results. The image analysis was performed according to Node-RADS recommendations, guided by a three-level flowchart (Figure 1) [19]. The assessment categories considered were the size and configuration criteria and each subcategories score, with an overall score defining the likelihood of a nodal invasion on a scale from 1 to 5 (1: very low; 2: low; 3: equivocal; 4: high; 5: very high). Specifically, a template including lymph node packets from the true pelvis (bilateral hypogastric, obturator, and external iliac) and bilateral common iliac nodes were considered. Each packet was scored separately and matched to its final pathological exam.

### 2.4. Pelvic Lymph-Node Dissection and Pathologic Assessment

Bilateral extended PLND, in addition to RC, was performed by one of two experienced surgeons (C.L. and M.G.). Extended PLND typically refers to the removal of nodes between the aortic bifurcation and common iliac vessels proximally, the genitofemoral nerve laterally, the circumflex iliac vein distally, and the internal iliac vessels posteriorly [21]. A specific submission technique for PLND consisted of submitting separate packets of a lymph node from the true pelvis (bilateral hypogastric, obturator, and external iliac) and bilateral common iliac nodes. Specimens were evaluated according to our institution’s standard protocol. Lymph nodes were processed by a standard method that included the dissection of nodes away from the adipose tissue under bright light. No fat-clearing solutions were used. All identified nodes were sectioned and pathologically evaluated. LNI was defined as the presence of ≥1 positive node among those removed and pathologically evaluated. The LNI status was assessed for each lymph node packet separately. Moreover, an overall LNI status for each patient was reported according to pathologic N stage (pN0 s pN ≥ 1).

### 2.5. Statistical Analyses

All analyses were run both at the patient-level and at the lymph node level. In the first scenario, we considered for each patient, the highest Node-RADS score assigned by the radiologist and matched with final pathology report (pN0 s pN ≥ 1). In the second scenario, we matched the Node-RADS score to the corresponding pathology for each lymph node packet separately.

First, to test for trends in the LNI rates, according to the Node-RADS score, ranging from one to five, a Cochran Armitage test was performed. Second, we fitted univariable and multivariable logistic regression models to test the association between the Node-RADS score and LNI. Multivariable adjustments were made for age, cT stage, and concomitant CIS at the last TURBT. Since only one patient harbored LVI in the last TURBT, we decided to exclude this covariate from the multivariable model, based on statistical considerations. Furthermore, a Harrel C-index was computed for the full model, as well as for a restricted model, excluding the Node-RADS score, to examine its relative contribution to estimate the LNI risk. Subsequently, a local polynomial smoother function (LOESS) [22,23] depicted the predicted probability of LNI, according to the Node-RADS score, after accounting for all of the above-mentioned confounders. Then, the receiver operating characteristic (ROC) and the area under the curve (AUC) depicted the diagnostic performance of the Node-RADS score for LNI. Finally, the sensitivity, specificity, positive predicted value (PPV), and negative predicted value (NPV) were estimated for all possible cut-offs of the Node-RADS score (>1 vs. >2 vs. >3 vs. >4).

All analyses were repeated both at the patient and lymph node levels. All tests were two-sided with a level of significance set at *p* < 0.05 and the R software environment for statistical computing and graphics (version 3.4.3) was used for all analyses.

## 3. Results

### 3.1. Study Population Characteristics

Overall, 49 BCa patients treated with RC plus bilateral extended PLND were eligible for this study (Table 1). The median age was 70 years (IQR 61–77) and 75.5% of patients were males. According to the preoperative staging, most patients harbored organ confined disease (cT ≤ 2, 59.2%). Conversely, only a minority of patients exhibited the presence of LVI (2.0%) or concomitant CIS (12.2%) in the last TURBT. Concerning the treatment options, an open RC was performed in most of the patients, in comparison with a minimally invasive approach (77.6 vs. 22.4%, respectively). The median number of the lymph node removed was 21 (IQR 15–26) and a total of 396 lymph node packets were identified.

### 3.2. Lymph Node Invasion Rates According to the Node-RADS Score

**Figure 2:**
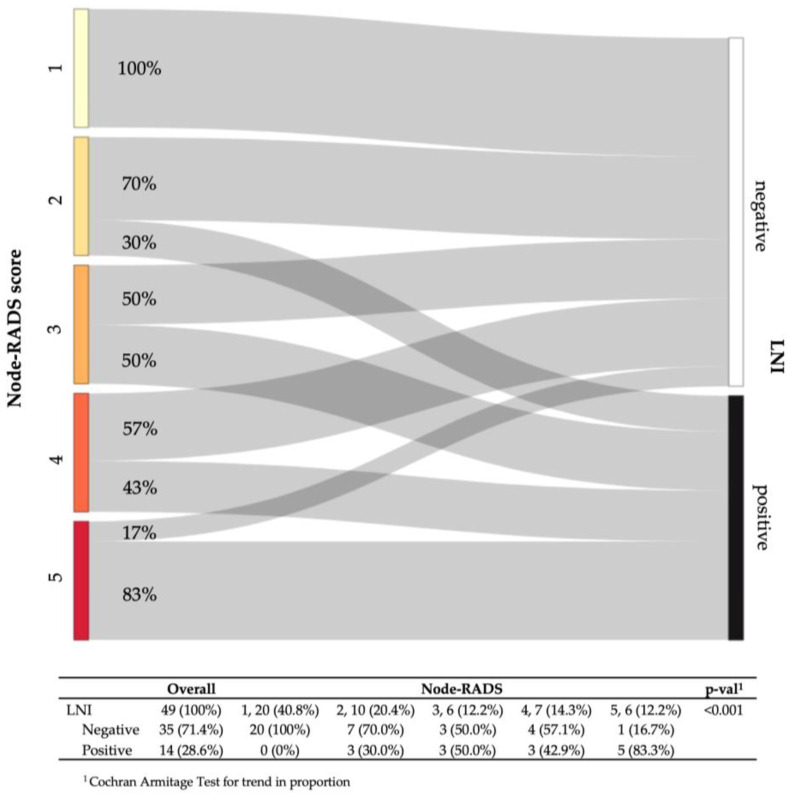
Sankey diagram depicting the lymph-node invasion (LNI) rates at the final pathologic examination, according to the preoperative Node-Rads score at the patient-level.

At a patient level, the overall LNI rate was 29% (14/49). Based on the blinded CT image evaluation, 20 (40.8%) vs. 10 (20.4%) vs. 6 (12.2%) vs. 7 (14.3%) vs. 6 (12.2%) of patients were assigned a Node-RADS score 1 vs. 2 vs. 3 vs. 4 vs. 5, respectively. Following the stratification according to the Node-RADS scores, the LNI rates ranged from 0 to 83.3% with increasing scores (*p* < 0.001) (Figure 2). At a lymph node level, the overall LNI rate was 8.7% (34/396). Based on a blinded CT image evaluation, 208 (53.1%) vs. 134 (34.2%) vs. 22 (5.6%) vs. 12 (3.1%) vs. 16 (4.1%) of patients were assigned a Node-RADS score 1 vs. 2 vs. 3 vs. 4 vs. 5, respectively. Following the stratification according to the Node-RADS Sscores, the LNI rates ranged from 0 to 75.0% with increasing scores (*p* < 0.001) (Supplementary Table S1).

**Figure 3:**
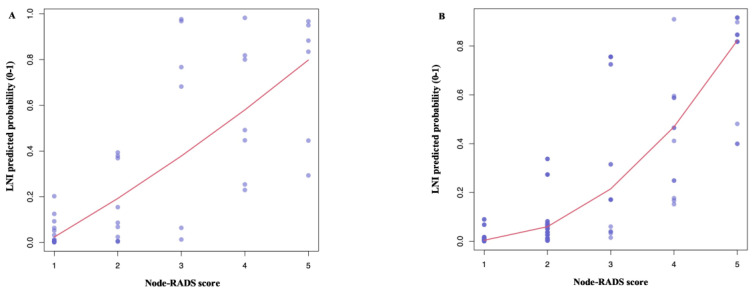
Local polynomial smoother function (LOESS) depicted the predicted probability of lymph-node invasion (LNI) according to the Node-RADS score at the patient level (**A**) and at the lymph node level (**B**). Predicted probability was derived from a multivariable logistic regression model accounting for important cofounders (age, cT stage, and concomitant CIS at last TURBT).

In a univariable logistic regression analysis, the Node-RADS correlated with LNI both at the patient (OR 2.87, 95%CI 1.69–5.60, *p* = 0.001) and lymph node levels (OR 4.52, 95%CI 3.19–6.78, *p* < 0.001). Following the multivariable adjustments for important confounders, Node-RADS was found to be an independent predictor of LNI, both at the patient (OR 3.38, 95%CI 1.68–9.40, *p* = 0.004, Supplementary Table S2A) and lymph node levels (OR 5.18, 95%CI 3.39–8.64, *p* < 0.001, Supplementary Table S2B). The full model (Node-RADS, age, cT and CIS) exhibited a higher accuracy than a model without Node-RADS, both at the patient (C-index: 0.94 vs. 0.86, respectively) and lymph node levels (C-index: 0.94 vs. 0.81, respectively). Additionally, an LOESS analysis depicted an increase in the LNI risk relative to the increasing Node-RADS score (Figure 3).

### 3.3. Diagnostic Performance of the Node-RADS Score According to a Different Cut-off

**Figure 4:**
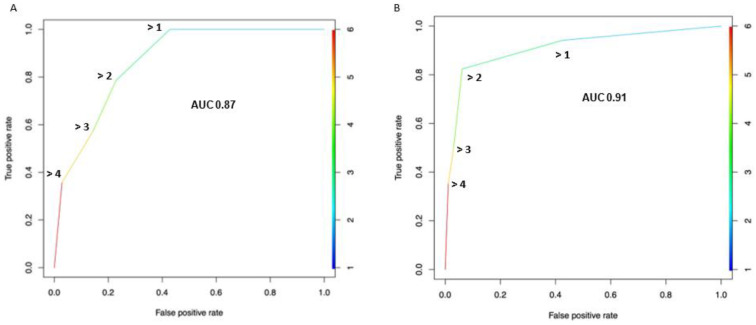
Receiver operating characteristic (ROC) curve depicting the diagnostic performance of the Node-Rads score for the detection of lymph-node invasion across all possible cut-offs, at both the patient (**A**) and lymph node levels (**B**). The area under the curve (AUC) was calculated and reported. True positive rate = sensitivity; false positive rate = 1 − specificity.

Based on the SROC curve, The AUC of the Node-RADS score was 0.87 at the patient level (Figure 4A) and 0.91 at the lymph node level (Figure 4B), respectively.

At the patient level, by setting a higher Node-RADS cut-off (from 1 to 5), the specificity and PPV increased from 57.1 to 97.1% and from 48.3 to 83.3%, respectively. Conversely, the sensitivity and NPV decreased from 100 to 35.7% and from 100 to 79.1%, respectively (Table 2). Similar trends were recorded at the lymph node level analysis (Supplementary Table S3). Interestingly, Node-RADS > 2 could be considered as the best cut-off, based on a balanced sensitivity and specificity at both the patient (78.6 and 77.1%, respectively) and lymph node levels (82.4 and 93.9%, respectively).

## 4. Discussion

Current cross-sectional imaging modalities are not accurate predictors of LNI, and the diagnostic performance can vary widely with the size criterium and general appearance being the most commonly used variables to suspect a nodal invasion [3,5]. Recently, Node-RADS scoring was introduced to provide a standardized comprehensive evaluation of the lymph node status, based on a five-item Likert scale accounting for both size and configuration criteria [19]. In the current study, we tested the overall diagnostic performances of the Node-RADS scores and hypothesized that the Node-RADS score independently correlates with LNI.

First, the demographic characteristics of our study cohort, such as median age and gender distribution, were concordant with the worldwide epidemiology of BCa [24,25]. Moreover, a LNI rate of 29% in the current study cohort reflected the 22% reported by Moschini et al. in a large multi-institutional cohort (*N* = 2778) [26]. Additionally, the number of lymph nodes removed, based on an extended template, as well as the proportions of concomitant CIS and LVI rates in the last TURBT, were comparable to results from a large Italian single center cohort with similar inclusion criteria [27]. Taken together, despite the limited sample size, our study cohort represents a reliable sample to provide a preliminary report for the potential role of Node-RADS in BCa patients.

Furthermore, we observed a positive trend in the LNI rates according to the Node-RADS scores. Node-RADS scores increased by three-fold the LNI risk and resulted in an independent predictor status after multivariable adjustments (OR 3.38, *p* = 0.004). This association was clearly depicted in the LOESS curve showing the progressive rise of the LVI risk in accordance with the increasing Node-RADS score. In addition, we observed a higher model accuracy when adding Node-RADS on top of other covariates (0.94 vs. 0.86), suggesting that the Node-RADS score contributes to accurately estimate the LNI risk. Similar results were obtained when considering each lymph node packet independently. Taken together, all of our findings pointed in the same direction, supporting the relevant association and the fundamental contribution of the Node-RADS score for the LNI assessment.

Node-RADS scores yielded a moderate-to-high classification accuracy (AUC 0.87) at the ROC curve analysis. Conversely, Moschini et al. reported a much lower classification accuracy (AUC 0.57), based on size criteria for the definition of the clinical N status [26]. In that study, only 7% of patients were considered cN+ and extreme values of sensitivity (18%) and specificity (96%) were recorded. Similarly, Hitier-Berthault et al. reported a 9% sensitivity vs. 90% specificity when implementing a 10 mm long axis cut-off definition [28]. In contrast, the Node-RADS score, which included both size and configuration criteria and provided a 5-item Likert scale response, allowed the ability to investigate the diagnostic performance at different cut-offs with a sensitivity ranging from 36 to 100% and a specificity from 57 to 97%. As such, the Node-RADS score may provide a more accurate and flexible tool for the identification of LNI in BCa patients. However, these results require further validation in a larger cohort with multiple readers and a direct comparison between Node-RADS and other accepted definitions of the clinical N status should be undertaken.

Lastly, a Node-RADS score of 2 could be considered as the best cut-off, based on the moderate to high balanced sensitivity and specificity values at both the patient level and lymph node level analyses. Interestingly, a Node-RADS 2 as cut-off would consider 30 out of 49 (61.2%) individuals as “negative” at the price of missing three patients with LNI within those 30 below the Node-RADS cut-off (10.0%), differently from other-RADS [29]. As a consequence, implementing this cut-off would result in considering around two-third of patients at a low risk of LNI (cN0) with an NPV of 90.0%. Nonetheless, it is important to underscore that the cut-off selection should rely on clinical considerations. Specifically, higher or lower cut-offs would provide a higher specificity with a lower sensitivity or vice versa. As such, further study should examine the Node-RADS cut-off according to the specific clinical scenarios, including the decision to perform PLND and its extension, the decision to administer neoadjuvant chemotherapy, and the evaluation of the chemotherapy response and subsequent consolidative local therapy. Specifically, urologists are now wondering whether to perform LND in all patients and with the same template. For example, Gschwend JE et al. failed to demonstrate any recurring free survival benefit for extended vs. limited LND in cT1G3 and cT2-cT4s non metastatic BCa patients treated with radical cystectomy [29]. It is possible that a better selection of patients, based on the preoperative risk of lymph node involvement, would deeply affect the results of such a trial. Moreover, European and North American guidelines recommend neoadjuvant chemotherapy followed by radical cystectomy (RC) only for cN negative muscle-invasive BCa patients, based on results from randomized clinical trials [7,8,9]. Conversely, management of cN positive BCa patients mainly relied on retrospective studies [10,11,12,13,14,15,16,17,18], suggesting systemic chemotherapy with addition of consolidative local therapy, in case of a favorable response [1,2]. Under such premises, an accurate and standardized assessment of the cN stage is strongly required to deliver optimal treatment and adequate counseling.

To the best of our knowledge, we are the first to test the diagnostic performance of Node-RADS in BCa patients treated with RC and extended PLND and as such, our results cannot be directly compared to other studies. Specifically, previous authors relied on multiple definitions while investigating the role of cross-sectional imaging in the cN staging of BCa [5]. The heterogeneity of their results has led urologists to progressively distrust cross-sectional imaging for detection of LNI in BCa patients [3]. Despite the limited sample size, our results suggested that Node-RADS may level these differences and provide a reproducible and reliable method for cN staging. Notably, we provided not only a patient-level analysis, as previous authors have provided [26,28], but also a lymph node level analysis, by which we further corroborated the ability of the Node-RADS score to identify positive nodes (AUC 0.91). Nonetheless, several questions remain open. First and foremost, we need to understand whether MRI and CT scans perform equally well in the Node-RADS score assignment. This question is of crucial importance since MRI has gained increasing popularity after the successful introduction of VI-RADS scoring for cT staging in BCa [30,31,32,33,34]. As a consequence, MRI might play a role as a cost-effective all-in-one strategy for comprehensive cT and cN staging of BCa patients at the initial diagnosis, as it has been demonstrated for prostate cancer [35,36,37,38,39]. Following the identification of the best imaging modalities, further analysis should investigate the Node-RADS cut-offs according to both specific clinical and research scenarios. For example, if our objective is to exclude patients without LNI while limiting false negatives, we will need to adopt a lower cut-off with a higher NPV. Conversely, if our objective is to identify patients with LNI while limiting the false positives, we will adopt a higher cut-off with a higher PPV. Finally, the Node-RADS score might be integrated within models predicting LNI. For example, Moschini et al. built a nomogram to predict the PLND extension (age, c T stage, cN stage, CIS, and LVI at last TURBT) and reported a C-index of 75% [26]. Here, the use of Node-RADS instead of the previous cN stage definition might help to improve the overall model accuracy. Moreover, either liquid biopsy and/or molecular biomarkers might improve the overall diagnostic and prognostic accuracy of Node-RADS itself, as it has been previously been carried out in other GU malignancies [40,41,42].

Despite the importance of our findings, our study is not devoid of limitations. First, our results relied on a limited sample size from a single institution. However, this represents a time and cost-effective study design to test the potentialities of the Node-RADS score. Moreover, when moving at the lymph-node level, we analyzed a much bigger sample of 396 lymph-node packet supporting patient-level findings. According to our promising results, further studies should be developed, including retrospective and/or prospective data from multiple institutions for results validation. Second, we used only CT scans for the evaluation of the Node-RADS score, since this represents the most widely adopted preoperative staging modality, although the increasing employment of MRI and VI-RADS scoring for bladder cancer local staging [43,44]. Indeed, MRI most likely would provide higher accuracy values for the nodal assessment and studies aimed at demonstrating the performance of an MRI-based Node-RADS assessment for bladder cancer staging are warranted. Lastly, we excluded patients treated with neoadjuvant chemotherapy, since it represented a potential bias for whom a more complex study design would have been needed. Nonetheless, we relied on a standardized surgical approach, and a centralized radiologic and pathology assessment to provide the most unbiased estimate of the Node-RADS ability to predict LNI, by directly comparing the preoperative imaging with the histopathology report at both the patient and lymph node levels.

## 5. Conclusions

The current study lays the foundation for the introduction of a Node-RADS scoring system for the regional lymph-node assessment in bladder cancer patients. Specifically, the Node-RADS score was found to be an independent predictor of LNI after a multivariable adjustment. Furthermore, the Node-RADS score exhibited a moderate-to-high overall accuracy for the LNI identification, with the possibility of setting different cut-offs according to specific clinical scenarios.

## Figures and Tables

**Table 1:** Descriptive characteristics of 49 bladder cancer patients treated with radical cystectomy plus extended pelvic lymph-node dissection without preoperative systemic chemotherapy at our institution, from January 2019 to June 2022.

Characteristic	Overall, *N* = 49
**Age (years),** median (IQR)	70 (61, 77)
**Sex** *n* (%)	
Female	12 (24.5)
Male	37 (75.5)
**Clinical T stage**, *n* (%)	
Organ-confined (cT ≤ 2)	29 (59.2)
Non-organ confined (cT > 2)	20 (40.8)
**Tumor Grade (WHO 2004)**, *n* (%)	
Low Grade	0 (0.0)
High Grade	49 (100.0)
**Concomitant CIS**, *n* (%)	6 (12.2)
**Presence of LVI**, *n* (%)	1 (2.0)
**Surgical approach**, *n* (%)	
Open	38 (77.6)
Minimally invasive	11 (22.4)
**N° removed lymph nodes**, median (IQR)	21 (15, 26)
**Pathologic T stage**, *n* (%)	
<2	11 (22.4)
2	11 (22.4)
3–4	27 (55.1)
**Pathologic N stage**, *n* (%)	
0	35 (71.4)
1	5 (10.2)
2	6 (12.2)
3	3 (6.1)
**Surgical positive margin**, *n* (%)	3 (6.1)

**Table 2:** Sensitivity, specificity, positive predictive value (PPV), negative predictive value (NPV), and accuracy were reported for the different Node-Rads cut-offs.

Node-Rads Cut-off	Pts above Cut-off *n* (%)	Pts. below Cut-off *N* (%)	Specificity (%)	Sensitivity (%)	NPV (%)	PPV (%)	Accuracy (%)
**>4**	6 (12.2)	43 (87.8)	97.1	35.7	79.1	83.3	79.6
**>3**	13 (24.5)	36 (75.5)	85.7	57.1	83.3	61.5	77.6
**>2**	19 (38.8)	30 (61.2)	77.1	78.6	90.0	57.9	77.6
**>1**	29 (59.2)	20 (40.8)	57.1	100.0	100.0	48.3	69.4

* Patients = Pts.

## Data Availability

The data presented in this study are available upon request from the corresponding author. The data are not publicly available due to restrictions.

## Supplementary Materials

The following supporting information can be downloaded at: https://www.mdpi.com/article/10.3390/cancers15030580/s1, Table S1: Lymph-node invasion (LNI) rates at final pathologic examination according to preoperative Node-Rads score for all lymph node packets analyzed (lymph node level).; Table S2: Multivariable logistic regression models addressing lymph-node invasion in bladder cancer patients treated with radical cystectomy plus extended pelvic lymph-node dissection at both patient-level (2A) and lymph-node level (2B). Node-RADS was considered as a linear predictor or categorized according to clinically meaningful cut-offs (3 or 4). Harrel C-index was computed for all models.; Table S3: Sensitivity, Specificity, Positive Predictive Value (PPV), Negative Predictive Value (NPV) and Accuracy were reported for different Node-Rads cut-offs.
