# Supplementary material for: Performance of Node-RADS Scoring System for a Standardized Assessment of Regional Lymph Nodes in Bladder Cancer Patients
Source: Cancers (Basel). 2023 Jan 18;15(3):580. doi: 10.3390/cancers15030580 (PMC9913205; doi:10.3390/cancers15030580)
Supplement: Supplementary file 1 [file cancers-15-00580-s001.zip › cancers-2111964-supplementary.pdf]

Supplementary Table S1. Lymph-node invasion (LNI) rates at final pathologic examination according to preoperative Node-Rads score for all lymph node packets analyzed (lymph node level).

	**Overall**	**Node-RADS**					**p-val[1]**
LNI	392 (100%)	1, 208 (53.1%)	2, 134 (34.2%)	3, 22 (5.6%)	4, 12 (3.1%)	5, 16 (4.1%)	<0.001
Negative	358 (91.3%)	206 (99.0%)	130 (97.0%)	11 (50.0%)	7 (58.3%)	4 (25.0%)	
Positive	34 (8.7%)	2 (1.0%)	4 (3.0%)	11 (50.0%)	5 (41.7%)	12 (75.0%)	

Supplementary Table S2. Multivariable logistic regression models addressing lymph-node invasion in bladder cancer patients treated with radical cystectomy plus extended pelvic lymph-node dissection at both patient-level (**2A**) and lymph-node level (**2B**). Node-RADS was considered as a linear predictor or categorized according to clinically meaningful cut-offs (3 or 4). Harrel C-index was computed for all models.

**2A** (Patient level)	**Model with Node-RADS** (Linear predictor)		**Model with Node-RADS** *(1-2 vs ≥ 3 vs)*		**Model with Node-RADS** *(1-2 vs ≥ 4)*		**Model without Node-RADS**	
	**OR (95% CI)**	**p-value**	**OR (95% CI)**	**p-value**	**OR (95% CI)**	**p-value**	**OR (95% CI)**	**p-value**
**Node-RADS** (*score*)	3.38 (1.68- 9.40)	0.004						
**Node-RADS**								
1-2			ref.					
≥ 3			9.50 (1.79-69.2)	0.013				
**Node-RADS**								
1-3					ref.			
≥ 4					16.88 (2.53-217.00)	0.009		
**Age** (*years*)	0.92 (0.81-1.02)	0.2	0.94 (0.85-1.03)	0.2	0.91 (0.80-1.01)	0.1	0.94 (0.86-1.02)	0.2
**Clinical T stage**								
Organ-confined (cT≤2)	ref.		ref.		ref.		ref.	
Non-organ confined (cT>2)	30.16 (3.20-729.00)	0.011	19.06 (2.82-251.72)	0.008	44.35 (4.93-1148.65)	0.004	19.86 (3.73-177.57)	0.002
**Concomitant CIS**								
Absent	ref.		ref.		ref.		ref.	
Presence	33.00 (1.58-2695.59)	0.058	12.90 (0.85-338.64)	0.086	24.14 (1.88-838.49)	0.032	13.69 (1.49-205.99)	0.032
**C-index**	0.94		0.91		0.92		0.86	
OR = odds ratio; CI = confidence interval, CIS = carcinoma in situ; NOC = Non-organ confined (cT>2); OC = Organ-confined (cT≤2)								

**2B**<br>(Lymph node level)	**Model with Node-RADS**<br>(Linear predictor)		**Model with Node-RADS**<br>(*1-2 vs ≥ 3 vs*)		**Model with Node-RADS**<br>(*1-2 vs ≥ 4*)		**Model without Node-RADS**	
	**OR (95% CI)**	**p-value**	**OR (95% CI)**	**p-value**	**OR (95% CI)**	**p-value**	**OR (95% CI)**	**p-value**
**Node-RADS** (*score*)	5.18 (3.38-8.64)	<0.001						
**Node-RADS**								
1-2			ref.					
≥ 3			54.09 (19.96-171.64)	<0.001				
**Node-RADS**								
1-3					ref.			
≥ 4					83.83 (24.83-350.00)	<0.001		
**Age** (*years*)	0.96 (0.90-1.02)	0.2	0.99 (0.93-1.04)	0.6	0.95 (0.88 -1.01)	0.083	0.99 (0.95-1.04)	0.8
**Clinical T stage**								
Organ-confined (cT≤2)	ref.		ref.		ref.		ref.	
Non-organ confined (cT>2)	10.68 (2.76-54.33)	0.002	8.62 (2.45-38.52)	0.002	25.78 (6.61-149.03)	<0.001	13.32 (4.94-46.79)	<0.001
**Concomitant CIS**								
Absent	ref.		ref.		ref.		ref.	
Presence	10.86 (3.40-38.95)	<0.001	4.40 (1.23-16.57)	0.024	17.02 (5.56 -58.44)	0.032	4.85 (2.01-11.59)	<0.001
**C-index**	0.94		0.93		0.94		0.81	

OR = odds ratio; CI = confidence interval, CIS = carcinoma in situ; NOC = Non-organ confined (cT>2); OC = Organ-confined (cT≤2)

Supplementary Table S3. Sensitivity, Specificity, Positive Predictive Value (PPV), Negative Predictive Value (NPV) and Accuracy were reported for different Node-Rads cut-offs.

NODE-RADS CUT-OFF	SPECIFICITY	SENSITIVITY	NPV	PPV	ACCURACY
> 4	98.9	35.3	94.1	75.0	93.4
> 3	96.9	50.0	95.3	60.7	92.9
> 2	93.9	82.4	98.2	56.0	92.9
> 1	57.5	94.1	99.0	17.4	60.7
